# Sclerostin induced tumor growth, bone metastasis and osteolysis in breast cancer

**DOI:** 10.1038/s41598-017-11913-7

**Published:** 2017-09-12

**Authors:** Menghai Zhu, Changzhen Liu, Shifei Li, Shudong Zhang, Qi Yao, Qingkun Song

**Affiliations:** 1 Department of Orthopedics, Peking University Ninth School of Clinical Medicine, Beijing Shijitan Hospital, Capital Medical University, Beijing, China; 2 Beijing Key Laboratory of Research of Chinese Medicine on Prevention and Treatment for Major Diseases, Experimental Research Center, China Academy of Chinese Medical Sciences, Beijing, China; 3 Department of science and technology, Beijing Shijitan Hospital, Beijing, China

## Abstract

Breast cancer is the second leading cause of cancer-related deaths among women worldwide. Many patients suffer from bone metastasis. Sclerostin, a key regulator of normal bone remodeling, is critically involved in osteolytic bone diseases. However, its role in breast cancer bone metastasis remains unknown. Here, we found that sclerostin was overexpressed in breast cancer tumor tissues and cell lines. Inhibition of sclerostin by antibody (Scl-Ab) significantly reduced migration and invasion of MDA-MB-231 and MCF-7 cells in a time- and dose-dependent manner. In xenograft model, sclerostin inhibition improved survival of nude mice and prevented osteolytic lesions resulting from tumor metastasis. Taken together, sclerostin promotes breast cancer cell migration, invasion and bone osteolysis. Inhibition of sclerostin may serve as an efficient strategy for interventions against breast cancer bone metastasis or osteolytic bone diseases.

## Introduction

Breast cancer is the second leading cause of cancer-related deaths among women worldwide. 75–80% of patients with advanced disease develop bone metastasis^1, 2^. As an end-stage complication of breast cancer, bone metastasis frequently leads to detrimental skeletal-related events associated with significant morbidity^3–5^. Since current treatment for bone metastasis has limited efficacy, development of an effective therapeutic strategy is urgently needed. To improve the prognosis of breast cancer bone metastasis (BCBM), molecular mechanisms involved in breast cancer proliferation, invasion, metastasis and osteolytic lesions should be further explored.

Sclerostin, a key regulator of normal bone remodeling, inhibits bone formation through inhibition of Wnt signaling^6, 7^. Recent studies have revealed a vital role of sclerostin in multiple myeloma with osteolytic bone lesions^8–12^. BCBM patients exhibit a high level of circulating sclerostin, which correlates with disease stage and fractures, however, the origin and impact of sclerostin on BCBM remains to be defined. Thus, the purpose of our study was to evaluate the expression level of sclerostin in tumor tissue derived from BCBM patients and explore its association with clinical outcome and tumor characteristics, including the presence of lytic bone disease.

Activation of Wnt signaling pathway has been indicated to participate in both initiation and progression of cancer metastasis^13, 14^. During multiple-steps of metastasis to bone, breast cancer cells successfully induce a sequence of changes, for instance, secreting cytokines to inhibit differentiation and maturation of osteoblasts whereas to enhance the activity of osteoclasts. As such, we hypothesized that decreased sclerostin would suppress osteolytic bone lesions and reduce tumor burden, which may represent a target for inhibiting cancer-induced bone diseases and facilitating restoration of normal bone homeostasis.

## Results

### Sclerostin is up-regulated in tumor tissues derived from patients with BCBM

**Figure 1:**
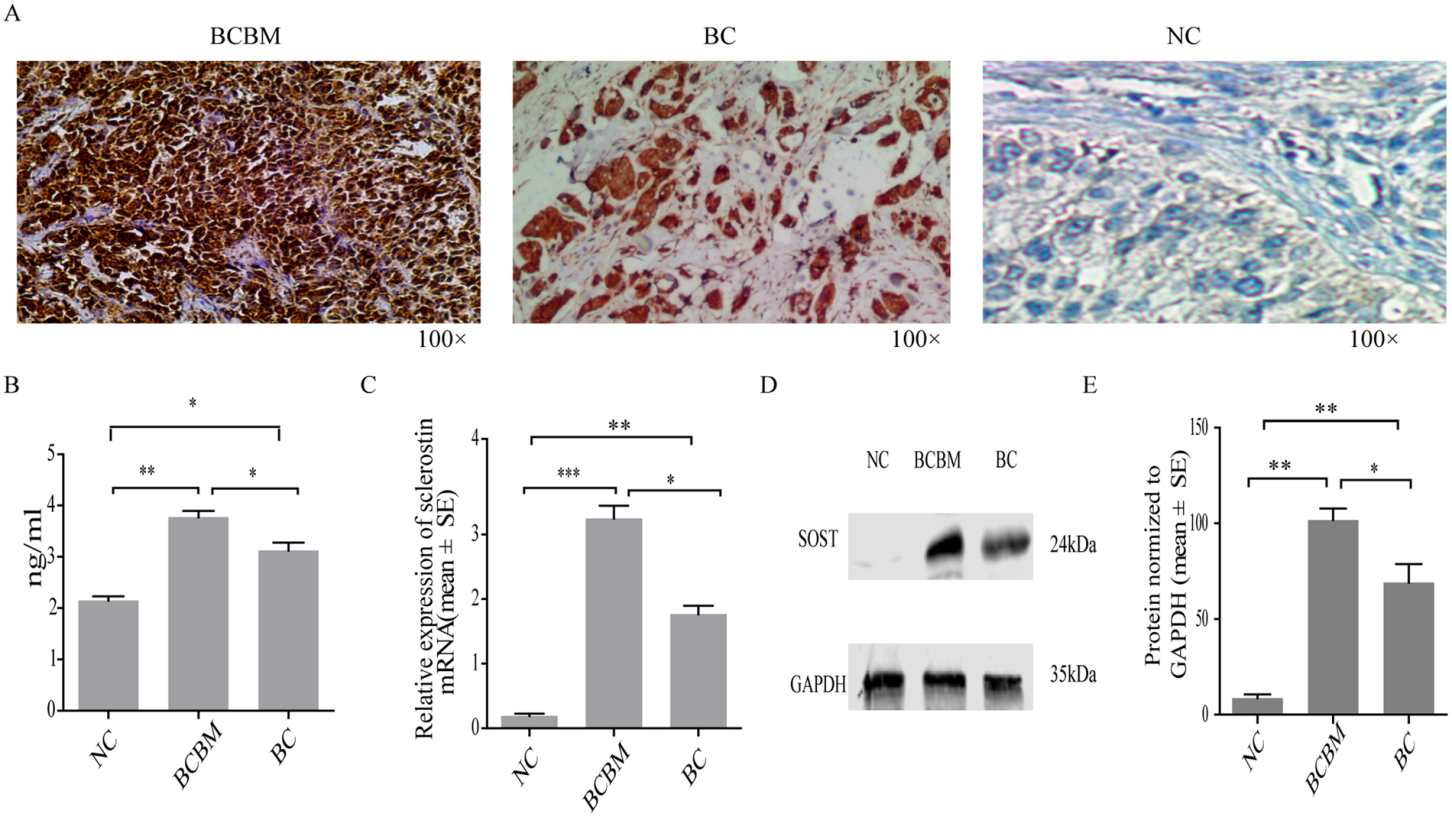
Expression of sclerostin (SOST) in tumor tissues. (**A**) Representative expression of sclerostin by immunohistochemical staining (100 × magnification). Left: strongly positive expression of sclerostin in breast cancer bone metastasis (BCBM) tumor cells; middle: weakly positive expression of sclerostin in localized breast cancer (BC); right: negative expression of sclerostin in benign breast tumor (NC) tissue. (**B**) Quantification of sclerostin in the plasma of NC, BC and BCBM patients by ELISA. Expression of sclerostin mRNA (**C**), protein (**D**) and protein (**E**) were quantified in NC, BCBM and BC tissues. Each bar represents mean ± SEM. ^*^P < 0.05, ^**^P < 0.01, ^***^P < 0.005.

To assess the expression of sclerostin in BCBM, paraffin sections of tissues from BCBM, localized breast cancer and benign breast tumor (n = 15 per group) were evaluated with immunohistochemistry (Fig. 1A). Among the tumor tissues from BCBM, 13 (86.7%) exhibited strongly positive expression of sclerostin. In breast cancer patients, mainly in the cytoplasm of tumor cells, 12 (80%) of samples were weakly positive for sclerostin. In contrast, all tissues from benign breast tumor were negative. We next quantified sclerostin in the plasma of breast cancer patients and matched health individuals by ELISA (Fig. 1B). Significantly upregulated expression of sclerostin was observed in BCBM compared with localized breast cancer and benign breast tumor (Fig. 1C), which was further confirmed by Western blot analysis (Fig. 1D).

### Expression of sclerostin in breast cancer cell lines

**Figure 2:**
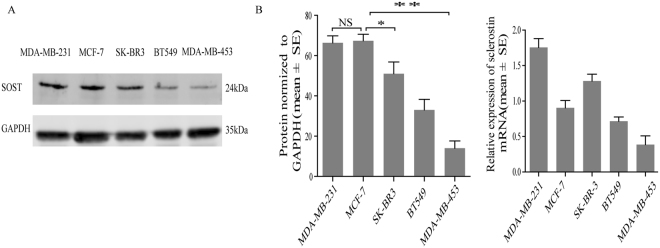
Expression of sclerostin in human breast cancer cell lines. (**A**)Expression of sclerostin protein. GAPDH was used as an internal control. Sclerostin protein was extracted from supernatant culture medium after centrifugation and concentration. (**B**) Quantification of sclerostin are normalizing to GAPDH. (**C**) Expression of sclerostin mRNA (RT-PCR: 45 cycles of amplification). GAPDH mRNA was used as an internal control. Each bar represents mean ± SEM.

To determine the expression profile of sclerostin in different breast cancer cell lines (MDA-MB-231, BT-549, MCF-7, MDA-MB-453 and SK-BR3), qRT-PCR assay and Western blot analysis were adopted. Sclerostin protein extracted from supernatant culture medium was detectable in all cell lines (Fig. 2A). A higher expression level was observed in MDA-MB-231 and MCF-7 cells, whereas a lower level in SK-BR3, BT-549 and MDA-MB-453 cells. However, concerning expression of sclerostin mRNA, SK-BR3 was higher than MCF-7 (Fig. 2B).

### Effect of sclerostin on migration and invasion of MDA-MB-231 and MCF-7 cells

**Figure 3:**
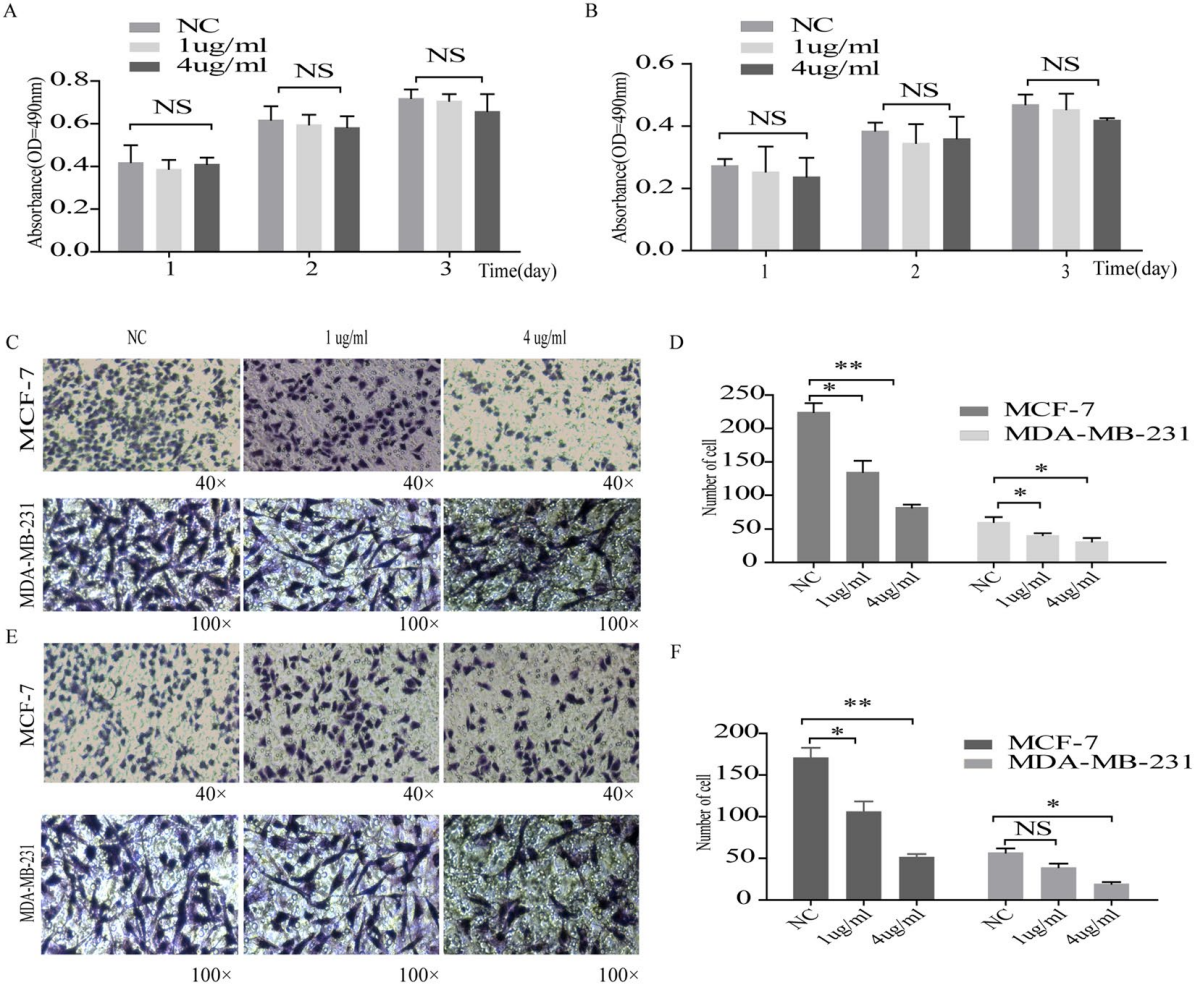
Effect of sclerostin inhibition on cell proliferation, migration and invasion. MTT assays were performed at 1d, 2d, and 3d after treatment with sclerostin antibody (at 0, 1 and 4 µg/ml, respectively) in (**A**) MCF-7 and (**B**) MDA-MB-231 cells. (**C**) Cell migration assays were performed using Transwell system. MDA-MB-231 and MCF-7 cells were treated with sclerostin antibody (at 1 µg/ml and 4 µg/ml, respectively) for 24 h. Migrated cells were stained with crystal violet and (**D**) migration capability was evaluated by counting migrated cells. (**E**) Invasion assays were performed and (**F**) invasiveness was quantified by counting invading cells. NC, non-treated control group; 1 µg/ml, 1 µg/ml sclerostin antibody-treated group; 4 µg/ml, 4 µg/ml sclerostin antibody-treated group. Each bar represents mean ± SEM. *P < 0.05, **P < 0.01. All experiments were performed at least three times with duplication within each individual experiment.

To explore the impact of reduced sclerostin expression on migration and invasion, breast cancer cell lines with high endogenous sclerostin were treated with blocking antibody against sclerostin. A subsequent MTT assay revealed that reduced sclerostin did not affect cell proliferation (Fig. 3A,B). Meanwhile, the migratory ability of cells was inhibited (Fig. 3C,D). Invasiveness of breast cancer cell lines treated with Sci-Ab was significantly undermined compared with control as demonstrated by Transwell chamber assay (Fig. 3E,F). These observations indicated that decreased sclerostin may suppress migration and invasion of breast cancer cells.

### Effect of sclerostin inhibition on tumor growth *in vivo*

**Figure 4:**
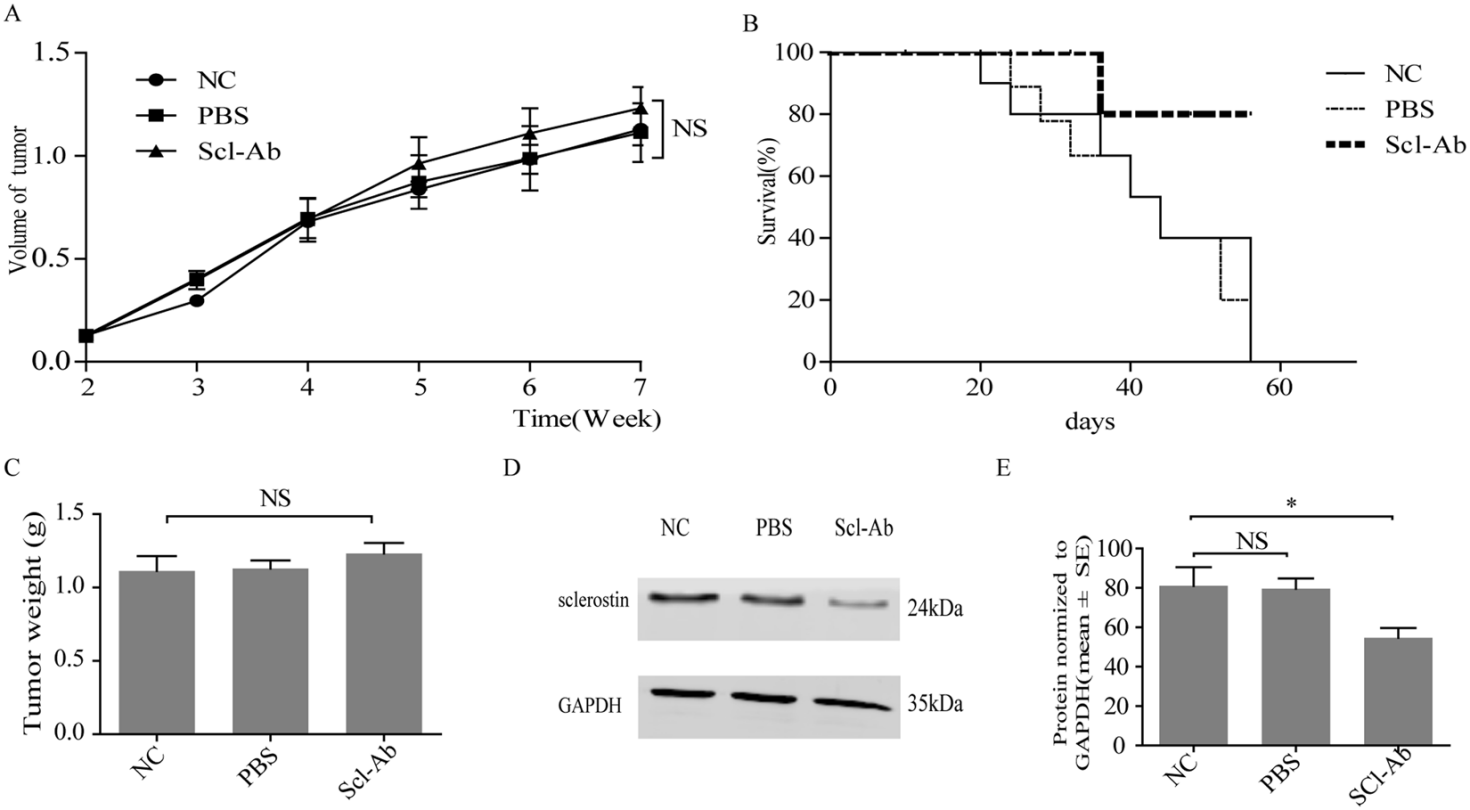
Effect of Scl-Ab on tumor growth *in vivo*. A breast cancer xenograft model was established using MDA-MB-231 cells (5 × 10^6^ cells in 30 μl PBS) implanted into the femur of female nude mice (7–8 weeks old). (**A**) Tumor size was measured using Vernier caliper once a week until the animals were sacrificed after 40 days of treatment. (**B**) Tumor weight was measured at the last time point. (**C**) Kaplan-Meier survival plot of xenografted control, PBS-treated and Sci-Ab treated groups. (**D**) Sclerostin protein expression and quantification (**E**) in tumor tissues of the above three groups. NC, non-treated control group; PBS, PBS-treated group; Sci-Ab, sclerostin antibody-treated group (1 μg antibody in 20 μl PBS). Each bar represents mean ± SEM. NS, non-significant.

**Figure 5:**
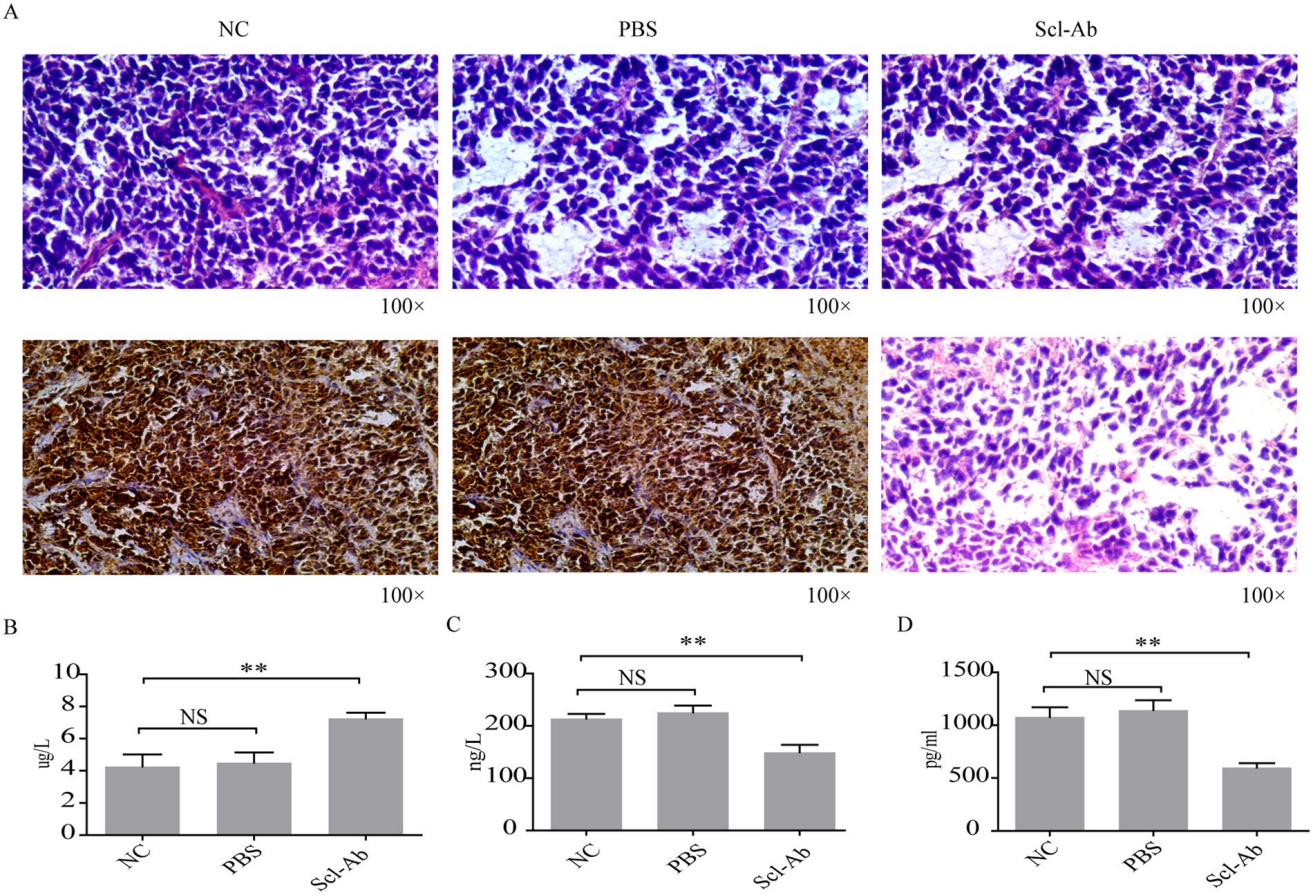
Analyses of tumor characteristics and serum of xenografted mice. (**A**) Representative H&E staining and immunohistochemistry for sclerostin (100 × magnification) in tumor sections obtained from different groups. (**B**) Quantification of osteocalcin (OCN), (**C**) osteoprotegerin (OPG), and (**D**) sclerostin in serum of mice from different groups by ELISA. NC, non-treated control group; PBS, PBS-treated group; Scl-Ab, sclerostin antibody-treated group (1 μg antibody in 20 μl PBS). Each bar represents mean ± SEM. **P < 0.01, NS, non-significant.

To investigate whether suppression of sclerostin may lose growth advantage *in vivo*, MDA-MB-231 cells (5 × 10^6^ in 30 μl PBS) were injected into the bone marrow space of BALB/c-nu/nu mice and tumor volume was measured starting at 1 week later. Tumors from control and PBS-treated groups were comparable in volume and weight to those from Scl-Ab treated group (Fig. 4A,C). However, a survival curve indicated Scl-Ab-treated animals lived longer (Fig. 4B). Then, Scl-Ab treated mice exhibited reduced expression of sclerostin protein in tumor tissue as shown by western blot analyses (Fig. 4D,E). Tumors were confirmed by H&E staining and tumor sections were stained immunohistochemically to determine the expression of sclerostin. Notably, Scl-Ab treated group demonstrated a lower expression of sclerostin (Fig. 5A). Compared with controls, a higher level of osteocalcin (Fig. 5B) and a lower level of OPG (Fig. 5C) were identified in Scl-Ab treated group. Finally, a lower level of sclerostin in serum was detected in Scl-Ab treated mice (Fig. 5D), suggesting that reduced sclerostin expression correlates with a better survival.

### Micro-CT analysis *in vivo*

**Figure 6:**
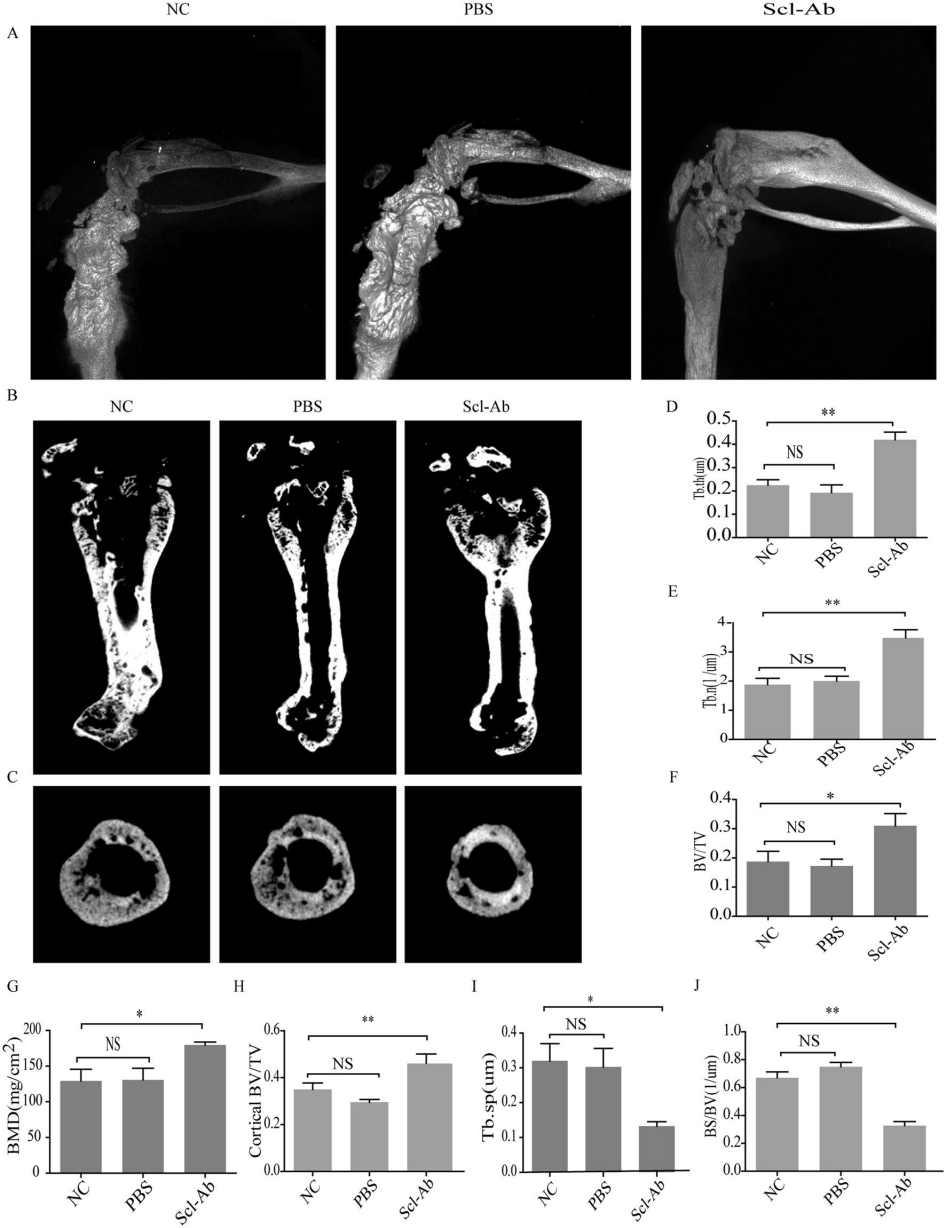
*In vivo* micro-CT analysis. (**A**) Presence of tumor-induced osteolytic lesions detected by micro-CT scans. Representative 3-D reconstruction of micro-CT images of femurs from non-treated control, PBS- and Sci-Ab treated mice. Three-dimensional images reconstructed from micro-CT analysis on the cortical and trabecular bone microarchitecture of whole left femur (**B**, longitudinal section) and of distal femoral metaphysis (**C**, cross section) in different groups. On Micro-CT, NC and PBS mice showed increased (**D**) trabecular thickness (Tb.Th), (**E**) trabecular number (Tb.N), (**F**) bone volume/tissue volume ratio (BV/TV), (**G**) bone mineral density (BMD), and (**H**) cortical bone volume/tissue volume ratio (cortical BV/TV), whereas decreased (**I**) trabecular separation (Tb.Sp) and (**J**) bone surface/bone volume ratio (BS/BV) compared with Scl-Ab treated mice. NC, non-treated control group; PBS, PBS-treated group; Scl-Ab, sclerostin antibody-treated group (1 μg antibody in 20 μl PBS). Each bar represents mean ± SEM. *P < 0.05, **P < 0.01; NS, non-significant.

In this study, micro-CT was performed to explore the presence of micro-structure of femoral bone in mice, and to evaluate sclerostin induced bone osteolysis in breast cancer. Furthermore, potential mechanism underlying osteolysis was investigated through micro-structure. In both control and PBS-treated animals, parameters of trabecular bone after 3-D reconstruction demonstrated severe impairment of micro-structure (Fig. 6A–C). Further, trabeculae became thinner and less dense (Fig. 6D and E) in those groups compared with Scl-Ab treated group. Compared with animals in control and PBS-treated groups, Scl-Ab treated animals exhibited higher bone volume/tissue volume ratio (BV/TV), trabecular thickness (Tb.Th), and bone mineral density (BMD) (Fig. 6F–H), whereas smaller inter-trabecular spaces (Fig. 6I) and lower BS/BV (Fig. 6J), indicating Scl-Ab may protect bone from breast cancer cells mediated damages.

## Discussion

Almost 80% of breast cancer patients have evidence of bone loss at baseline^15^ and 65–75% of patients suffer from metastases to bone^16^. Breast cancer-related bone disease results from aberrant activation of osteoclasts and impaired function of osteoblasts^17, 18^. A better understanding of risk factors and signaling pathways involved in the development and progression of BCBM may provide new biomarkers for early detection of metastasis and, eventually, improve patients’ quality of life. Sclerostin, a regulator of normal bone remodeling, reduces bone formation by inhibiting Wnt signaling. Although several facets of function have been discovered, pathologic role of sclerostin in bone metastasis remain undefined.

In this study, we observed a significantly increased level of sclerostin in the plasma of patients with breast cancer osseous metastasis compared with that of localized breast cancer and breast benign tumor. Increased expression of sclerostin was detected in breast cancer tumor tissues and cell lines. Scl-Ab suppressed migration and invasion of MDA-MB-231 and MCF-7 cells. In a xenograft nude mouse model, inhibition of sclerostin significantly improved overall survival of mice. Importantly, suppressed sclerostin prevented osteolytic lesions resulting from tumor metastasis *in vivo*. Our data suggest the role of sclerostin in breast cancer cell migration, invasion and metastasis to bone.

Aberrant activation of Wnt/β-catenin signaling contributes to tumor development and progression^19–24^. Wnt/β-catenin signaling is involved in bone homeostasis and regeneration^22^. A high level of circulating sclerostin, a potent inhibitor of osteoblastogenesis, was detected in patients with BCBM. Sclerostin gene expression is activated in multiple myeloma and associated with carcinogenesis, disease progression, and prognosis of cancer patients^8, 12^. As previous reported^10^, increased sclerostin secretion by myeloma cells can suppress the function of osteoblasts. In our study, high expression of sclerostin is associated with the severity of osteolytic lesions. Moreover, Scl-Ab treatment prolongs survival of nude mice.

Pathological changes caused by BCBM include growth condition of trabecular bone, bone mineralization, micro-injury, and abnormal morphology such as decreased volume of both cortical and trabecular bones^25, 26^. Development of micro-CT technique has contributed to a better understanding of micro-architecture of cancer-induced bone diseases. A recent study by Florio *et al*.^27^ used a bispecific heterodimeric antibody targeting sclerostin and DKK-1 contributing to bone formation, bone mass and bone strength in intact bones in mice, and fractured bones in rats. Our data demonstrate that Sci-Ab may prevent osteolytic lesions and prevent development of breast cancer bone disease. Furthermore, Scl-Ab suppressed tumor growth, supporting the possibility that significantly reduced migration and invasion of MDA-MB-231 and MCF-7 cells. Consistent with previously studies^25, 26^, a higher number of trabeculae, thinner inter-trabecular space, and more integral connectivity of the trabecular bone structure were identified in Scl-Ab-treated mice. Increased BV/TV, Tb.N., Tb.Th. and BS/BV values may result from reduced expression of sclerostin in bone. Moreover, Tb.Sp. and BS/BV were significantly lower in controls than in the Sci-Ab treated group, suggesting a positive therapeutic effect of Scl-Ab on cancer-induced bone destruction. This indicates that increased bone mass potentially results from recovered function of Wnt/β-catenin^28–31^. Consistent with precious reports^32–34^, a lower level of OPG (p < 0.01) and a higher level of osteocalcin (p < 0.01) were detected in Scl-Ab treated group compared with controls. However, further experiments are required to determine how Scl-Ab mechanistically affects secretion of OPG and OCN involved in cancer induced bone loss.

In conclusion, we have identified increased secretion of sclerostin by breast cancer cells. Inhibition of sclerostin by Scl-Ab suppresses migration and invasion of breast cancer cells. Furthermore, Scl-Ab may prevent osteolytic lesions as demonstrated by BCBM murine model bearing tumors generated by human breast cancer cells. Our study paves a way for further investigation into potential therapeutic targets for cancer-induced bone diseases.

## Methods

### Patients

This study investigated surgical resection samples from 15 patients with BCBM, as well as localized breast cancer and benign breast tumor (n = 15 per group). All patients were diagnosed and treated at Beijing Shijitan Hospital (Beijing, China). This study was approved by Institutional Review Boards at Beijing Shijitan Hospital. Informed consent was obtained from all participants and all methods were performed in accordance with the relevant guidelines and regulations.

### Cell Culture and Treatments

Breast cancer cell lines MCF-7 and MDA-MB-231 were obtained from Central Laboratory of Beijing Shijitan Hospital and cultured in Dulbecco’s Modified Eagle Medium (DMEM) containing 10% fetal bovine serum (FBS; Hyclone, GE Healthcare, Marlborough, MA), 50 units/ml of penicillin/streptomycin (Thermo Fisher Scientific, Waltham, MA), and puromycin (Sigma-Aldrich, St. Louis, MO). SK-BR3 cell line was obtained from Shanghai Cell Biological Institute of the Chinese Academy of Science (Shanghai, China) and cultured in DMEM supplemented with 10% FBS. BT-549 (cultured in RPMI-1640 Medium with 10% FBS and 0.023 IU/ml insulin) and MDA-MB-453 (maintained in Leibovitz’s L-15 Medium and 10% FBS with no CO_2_) cell lines were obtained from Central Laboratory of Peking University Health Science Center (Beijing, China). Except MDA-MB-453, all cell lines were incubated at 37 °C in a humidified-atmosphere containing 5% CO_2_.

### RNA isolation and qRT-PCR amplification

Total RNA was extracted from frozen and paraffin-embedded samples using TRIzol® reagent (Thermo) according to the manufacturer’s instructions. RNA from MDA-MB-231 and MCF-7 cells was reverse-transcribed using SuperScript® cDNA Synthesis Kit (Thermo). In brief, an RT mixture containing 1 μl total RNA, 4 μl dNTPs, 2 μl Primer Max, 4 μl RT buffer, 1 μl SuperRT, and diethyl pyrocarbonate (DEPC)-treated water to a final volume 20 μl was prepared according to the manufacturer’s instructions. Next, cDNA was transferred into a 20-μl PCR reaction mixture using 2× UltraSYBR Mixture (Beijing Cowin Biotech, Bejing, China). Reactions were performed in a 20-μl reaction volume consisting of 0.5 μl cDNA, 0.4 μl each of forward and reverse primers, 10 μl SYBR® Green I Master Mix (Applied Biosystems, Foster City, CA) and DEPC-treated water to a final volume of 20 μl. Primers were as follows: GAPDH forward 5′-GAAGGTGAAGGTCGGAGTC-3′ and reverse 5′-GAAGATGGTGATGGGATTTC-3′; sclerostin forward 5′-AAATCACATCCGCCCCAACT-3′ and reverse 5′-GGCGGTGTCTCAAAAGGGAT-3′. Amplification and detection were performed using a 7500HT Fast Real-Time PCR system (Applied Biosystems) and the 2× UltraSYBR Mixture (Beijing Cowin Biotech) as follows: 15 min at 95 °C and 40 cycles of 15 s at 95 °C and 30 s at 60 °C. Data were analyzed with SDS Software version 2.0 (Applied Biosystems).

### Western blot analysis

Western blot analysis was performed using protein extracted from cell lysates or supernatant culture medium, which were dissolved in loading buffer (5× solution of 50% glycerol, 10% sodium dodecyl sulfate, 5% β-mercaptoethanol, 0.5% bromophenol blue, and 250 mM Tris-HCl pH 6.8) and denatured for 5 min at 100 °C prior to electrophoresis. Proteins were analyzed using an 8–10% polyacrylamide gel and mid-range protein ladder (Beijing Cowin Biotech). Proteins were transferred for 90 min to polyvinylidene difluoride membranes using Bio-Rad semi-dry transblotters (Hercules, CA) and electroblotting (300 mA). Membranes were blocked for 1 hour at room temperature with Bovine Serum Albumin Blocking Buffer (Beijing Cowin Biotech). Strips were subjected to rabbit anti-sclerostin (1:1000; ab63097, Abcam, Cambridge, UK), rabbit anti-GAPDH (1:1000; EPR6256, ab128915, Abcam) antibodies over night at 4 °C. After incubation with polyclonal goat anti-rabbit IgG H&L Alexa Fluor® 790 (1:10000; Abcam), specific reactions were revealed with LI-COR’s Odyssey Infrared Imaging System and quantified by Odyssey 3.0 analytical Image Studio software (LI-COR Biotechnology, Lincoln, NE).

### 3-(4,5-dimethylthiazol-2-yl)-2,5-diphenyltetrazolium bromide (MTT) assay

MTT assay was conducted to evaluate inhibitory effect of sclerostin antibody (Scl-Ab, obtained from Central Laboratory at People’s hospital of Peking university) on proliferation of breast cancer cells. MDA-MB-231 and MCF-7 cells were seeded into 96-well plates with the density of 6 × 10^3^ cells/well and then incubated for 12–24 hours. Next, sclerostin antibody (at a concentration of 0, 1 and 4 µg/ml, respectively) was added to cells and incubated at 37 °C for 5–7 days. At the end of treatment, 20 μL of MTT solution (5 mg/ml) was added into each well and then plates were incubated for an additional 4 hours. After removing the medium, 160 μL/well of DMSO was added to each well. The optical density (OD) value of each well was measured spectrophotometrically at 490 nm.

### Migration assay

Migration assays were performed in 48-well Transwell® plates (8.0-μm pore size; Costar, Corning Incorporated, Corning, NY). After treatment with 1 or 4 µg/ml sclerostin antibody (Scl-Ab) for 48 hours, breast cancer cells from control and treatment groups were seeded into the upper chamber of Transwell system at a concentration of 5 × 10^3^ cells/well in 100 μl medium containing 5% FBS. The lower chamber was filled with 500 μl medium containing 10% FBS. After 24 hours of incubation at 37 °C with 5% CO_2_, the upper surface of each filter was carefully washed with phosphate-buffered saline (PBS) and the remaining cells were removed with a cotton wool swab. Cells that had migrated to the bottom side of Transwell membrane inserts were fixed with 4% paraformaldehyde and stained with crystal violet. Migrated cells were counted (3 wells per group, 5 central fields per Transwell) at 100 × magnification using an inverted microscope (FM-600, Shanghai Puda Optical Instrument, Shanghai, China). At least five random fields of vision were counted per well to quantify migrated cells. The entire assay was repeated at least three times.

### Invasion Assay

The invasive capacity of cancer cells was confirmed *in vitro* using a Transwell chamber coated with Matrigel® (Becton Dickinson, Franklin Lakes, NJ), in accordance with the manufacturer’s recommendation. Sci-Ab (0, 1 or 4 µg/ml) was added to MDA-MB-231 and MCF-7 cells for 24 hours. After collection and resuspension, cell suspension (5 × 10^4^ cells/ml) of 50 ul was added into the upper chamber. Next, Transwell system was incubated for 1 day at 37 °C, and then cells were washed carefully with PBS to clear off those remaining on the upper side of Transwell membrane. Then, cells invaded to the bottom side of the membrane were fixed and stained with crystal violet. The entire experiment was conducted independently and repeated at least three times.

### Experimental murine model of breast cancer bone metastasis

BABL/C female mice (aged at 6–8 weeks, approximately weighed at 30 g) were obtained from Beijing Vital River Laboratory Animal Technology (Beijing, China) and housed in accordance with Institutional Animal Care and Use Committee (IACUC) guidelines. This study was reviewed and approved by IACUC at Beijing Shijitan Hospital. All experiments were conducted according to IACUC guidelines. Mice were housed for 1 week before they were anesthetized by intraperitoneal injection of chloral hydrate (10%, 0.1 ml/g). An incision was made in anesthetized mice along the right knee. The patellar tendon and muscle were split longitudinally to expose the distal femur. A surgical scalpel was used to drill a tiny hole on the cortex and MDA-MB-231 cells (5 × 10^6^ cells in 30 μl PBS) were injected into the bone marrow space through the hole with a 100-μl Hamilton microsyringe (Shanghai Linbo Scientific Instruments, Shanghai, China). Breast cancer cells resuspended in 30 μl PBS were slowly introduced into the marrow space to avoid extravasation. The needle was removed, the hole sealed with bone wax, the patellar tendon reapproximated, and the wound sutured.

Tumor growth was monitored every 3 days and tumor was weighed. Tumor volume was calculated by the equation V (mm^3^) = (a × b^2^)/2, where a is equal to the largest diameter and b is equal to the perpendicular diameter. When tumors reached a size of ~100 mm^3^ (20 days), mice were randomly distributed into three groups (12 mice per group). The first group was used as a blank control (non-treated). The second group was intratumorally administered 20 μl of PBS, and the third group was intratumorally treated with sclerostin antibody (1 μg antibody in 20 μl of PBS). These treatments were repeated once after 5 days.

### Micro-CT analysis

After animals were sacrificed, femoral condyles including the implants were harvested and excess tissue was removed and immediately fixed in 10% neutral-buffered formalin solution. Samples (n = 36) randomly selected from each group were used for high-resolution micro-CT (Inveon MMCT, Siemens, Munich, Germany) at 60 kV and 220 mA with a 0.5 mm aluminum filter. The scanning protocol required 440 exposures of 360° with 1500 ms per projection and an effective pixel width of 8.99 μm. Cobra software (Exxim, Pleasanton, CA) was used to reconstruct images with a down-sampling of one and a beam hardening correction. The Inveon Acquisition Workplace (Siemens) utilized a standard CT camera with a high resolution, 12-bit X-ray imaging detector with 2048 × 3072 pixels configured for mouse imaging. Three-dimensional (3-D) analysis and reconstruction of trabecular bone was performed on femurs by Inveon Research Workplace (Siemens). Consequently, constant regions of interest (ROIs) were drawn using a ROI tool at the region 0.8–2.6 mm to distal femoral metaphyses. Using ROI boxes of the same size, data analysis tools were used to calculate the trabecular bone compartment, which was manually delineated from the cortical bone. The following variables were determined: trabecular bone volume fraction (BV/TV), trabecular number (Tb.N; 1/μm), bone mineral density (BMD; gm/cm^2^), trabecular thickness (Tb.Th; μm), cortical bone volume/tissue volume ratio (cortical BV/TV), trabecular separation (Tb.Sp; μm) and bone surface/bone volume ratio (BS/BV).

### Enzyme-linked immunosorbent assay

Osteocalcin (OCN), osteoprotegerin (OPG) and sclerostin were detected in mice using enzyme-linked immunosorbent assay (ELISA) kits (Inova Diagnostics, San Diego, CA) in accordance with the manufacturer’s recommendations. ELISA plates (BD Biosciences, San Jose, CA) were coated for 2 h at 37 °C with 1 g/ml GRP78 in coating buffer. Then, plates were washed with PBS four times and blocked with PBS containing 2% skim milk overnight. On the following day, QUANTA Lite® reagents (Inova Diagnostics) were used to perform ELISA according to the supplier’s protocol. Plates were read at 405 nm on an fluorometer and all samples were analyzed simultaneously. Absorption was determined with an ELISA reader at 450 nm (550 Microplate Reader; Bio-Rad) and the results were expressed as mean ± SEM.

### Immunohistochemistry

Tissues were fixed in 10% neutral-buffered formalin and subsequently embedded in paraffin. Paraffin-embedded tissues were cut at 4 µm, deparaffinized with xylene, and rehydrated for further hematoxylin and eosin (H&E) or 3,3′-diaminobenzidine peroxidase (DAB) immunohistochemical staining. Briefly, following a brief proteolytic digestion and peroxidase blocking of tissue slides, slides were incubated overnight with primary antibodies against sclerostin (ab63097, Abcam) at a dilution of 1:100 at 4 °C, respectively. Visualization employed a Dako EnVision System (Dako Diagnostics, Zurich, Switzerland).

### Statistical analysis

Differences were compared between control and treatment groups were evaluated by one-way ANOVA followed by Bonferroni’s multiple comparison test using the Prism software package version 5.03 (GraphPad Software, La Jolla, CA). All data were expressed as mean ± SEM. A difference was considered statistically significant at a level of P < 0.05.

## Electronic supplementary material

Supplementary Information

## References

[1] Siegel R, Ward E, Brawley O, Jemal A (2011). Cancer statistics, 2011: the impact of eliminating socioeconomic and racial disparities on premature cancer deaths. CA: a cancer journal for clinicians.

[2] Jemal A (2011). Global cancer statistics. CA: a cancer journal for clinicians.

[3] Sathiakumar N (2012). Mortality following bone metastasis and skeletal-related events among women with breast cancer: a population-based analysis of U.S. Medicare beneficiaries, 1999-2006. Breast cancer research and treatment.

[4] Amir E (2012). Prospective study evaluating the impact of tissue confirmation of metastatic disease in patients with breast cancer. Journal of clinical oncology: official journal of the American Society of Clinical Oncology.

[5] Oster G (2013). Natural history of skeletal-related events in patients with breast, lung, or prostate cancer and metastases to bone: a 15-year study in two large US health systems. Supportive care in cancer: official journal of the Multinational Association of Supportive Care in Cancer.

[6] van Dinther M (2013). Anti-Sclerostin antibody inhibits internalization of Sclerostin and Sclerostin-mediated antagonism of Wnt/LRP6 signaling. PloS one.

[7] Qin, W. *et al*. Mice with sclerostin gene deletion are resistant to the severe sublesional bone loss induced by spinal cord injury. Osteoporosis international: a journal established as result of cooperation between the European Foundation for Osteoporosis and the National Osteoporosis Foundation of the USA, 10.1007/s00198-016-3700-x (2016). 10.1007/s00198-016-3700-x 27436301

[8] Eda H (2016). Regulation of Sclerostin Expression in Multiple Myeloma by Dkk-1: A Potential Therapeutic Strategy for Myeloma Bone Disease. Journal of bone and mineral research: the official journal of the American Society for Bone and Mineral Research.

[9] Wang XT (2014). Bone marrow plasma macrophage inflammatory protein protein-1 alpha(MIP-1 alpha) and sclerostin in multiple myeloma: relationship with bone disease and clinical characteristics. Leukemia research.

[10] Colucci S (2011). Myeloma cells suppress osteoblasts through sclerostin secretion. Blood cancer journal.

[11] Terpos E (2012). Elevated circulating sclerostin correlates with advanced disease features and abnormal bone remodeling in symptomatic myeloma: reduction post-bortezomib monotherapy. International journal of cancer.

[12] Brunetti G (2011). Sclerostin is overexpressed by plasma cells from multiple myeloma patients. Annals of the New York Academy of Sciences.

[13] Bilir B, Kucuk O, Moreno CS (2013). Wnt signaling blockage inhibits cell proliferation and migration, and induces apoptosis in triple-negative breast cancer cells. Journal of translational medicine.

[14] Cai J (2013). MicroRNA-374a activates Wnt/beta-catenin signaling to promote breast cancer metastasis. The Journal of clinical investigation.

[15] Futakuchi M, Fukamachi K, Suzui M (2016). Heterogeneity of tumor cells in the bone microenvironment: Mechanisms and therapeutic targets for bone metastasis of prostate or breast cancer. Advanced drug delivery reviews.

[16] Meng X (2016). Myeloid-specific TGF-beta signaling in bone promotes basic-FGF and breast cancer bone metastasis. Oncogene.

[17] Yamaguchi M (2014). Curcumin analogue UBS109 prevents bone loss in breast cancer bone metastasis mouse model: involvement in osteoblastogenesis and osteoclastogenesis. Cell and tissue research.

[18] Sawant A (2013). Myeloid-derived suppressor cells function as novel osteoclast progenitors enhancing bone loss in breast cancer. Cancer research.

[19] Huang J (2014). EphA2 promotes epithelial-mesenchymal transition through the Wnt/beta-catenin pathway in gastric cancer cells. Oncogene.

[20] Li X (2013). SOX2 promotes tumor metastasis by stimulating epithelial-to-mesenchymal transition via regulation of WNT/beta-catenin signal network. Cancer letters.

[21] Yamada N (2013). Tumor-suppressive microRNA-145 targets catenin delta-1 to regulate Wnt/beta-catenin signaling in human colon cancer cells. Cancer letters.

[22] Burgers TA, Williams BO (2013). Regulation of Wnt/beta-catenin signaling within and from osteocytes. Bone.

[23] Galliera E (2016). Wnt signaling pathway inhibitors as promising diagnostic serum markers of osteolytic bone metastasis. Journal of biological regulators and homeostatic agents.

[24] Kyvernitakis I (2014). Effect of aromatase inhibition on serum levels of sclerostin and dickkopf-1, bone turnover markers and bone mineral density in women with breast cancer. Journal of cancer research and clinical oncology.

[25] Geffre CP (2015). Combined micro CT and histopathology for evaluation of skeletal metastasis in live animals. American journal of translational research.

[26] Previdi S (2013). Combination of the c-Met inhibitor tivantinib and zoledronic acid prevents tumor bone engraftment and inhibits progression of established bone metastases in a breast xenograft model. PloS one.

[27] Florio,M. *et al*. A bispecific antibody targeting sclerostin and DKK-1 promotes bone mass accrual and fracture repair. NATURE COMMUNICATIONS **7**, doi:10.1038/ncomms11505.2015.09.029 (2016). 10.1038/ncomms11505 PMC4894982 27230681

[28] Zhang R (2013). Wnt/beta-catenin signaling activates bone morphogenetic protein 2 expression in osteoblasts. Bone.

[29] Zhou J (2012). Effects of pulsed electromagnetic fields on bone mass and Wnt/beta-catenin signaling pathway in ovariectomized rats. Archives of medical research.

[30] Lara-Castillo N (2015). *In vivo* mechanical loading rapidly activates beta-catenin signaling in osteocytes through a prostaglandin mediated mechanism. Bone.

[31] Zahoor M, Cha PH, Min do S, Choi KY (2014). Indirubin-3′-oxime reverses bone loss in ovariectomized and hindlimb-unloaded mice via activation of the Wnt/beta-catenin signaling. Journal of bone and mineral research: the official journal of the American Society for Bone and Mineral Research.

[32] Wanby P, Nobin R, Von SP, Brudin L, Carlsson M (2016). Serum levels of the bone turnover markers dickkopf-1, sclerostin, osteoprotegerin, osteopontin, osteocalcin and 25-hydroxyvitamin D in Swedish geriatric patients aged 75 years or older with a fresh hip fracture and in healthy controls. Journal of endocrinological investigation.

[33] Piemontese M, Xiong J, Fujiwara Y, Thostenson JD, O’Brien CA (2016). Cortical bone loss caused by glucocorticoid excess requires RANKL production by osteocytes and is associated with reduced OPG expression in mice. American journal of physiology. Endocrinology and metabolism.

[34] Bonfa AC, Seguro LP, Caparbo V, Bonfa E, Pereira RM (2015). RANKL and OPG gene polymorphisms: associations with vertebral fractures and bone mineral density in premenopausal systemic lupus erythematosus. Osteoporosis international: a journal established as result of cooperation between the European Foundation for Osteoporosis and the National Osteoporosis Foundation of the USA.

