# Supplementary material for: Sclerostin induced tumor growth, bone metastasis and osteolysis in breast cancer
Source: Sci Rep. 2017 Sep 12;7:11399. doi: 10.1038/s41598-017-11913-7 (PMC5595999; doi:10.1038/s41598-017-11913-7)
Supplement: Supplementary file 1 — Supplementary Information [file 41598_2017_11913_MOESM1_ESM.pdf]

Sclerostin induced tumor growth, bone metastasis and osteolysis in breast cancer

Menghai Zhu[1*], Changzhen Liu[2*], Shifei Li[3], Shudong Zhang[3], Qi Yao[3,1], Qingkun Song[4]

[1]Department of Orthopedics, Peking University Ninth School of Clinical Medicine, Beijing Shijitan Hospital, Beijing, China
[2]Beijing Key Laboratory of Research of Chinese Medicine on Prevention and Treatment for Major Diseases, Experimental Research Center, China Academy of Chinese Medical Sciences
[3]Department of Orthopedics, Beijing Shijitan Hospital, Capital Medical University, Beijing, China
[4]Department of science and technology, Beijing Shijitan Hospital, Beijing, China
[*]These authors contributed equally to this study.
Correspondence to: Qi Yao, email: yqjh2010@163.com; or Qingkun Song, email: songqingkun@aliyun.com

GAPDH

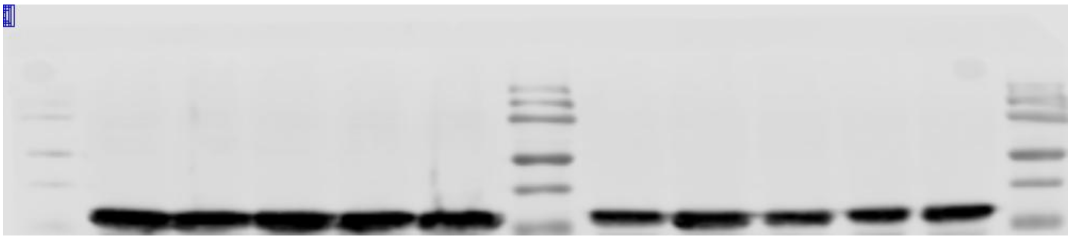

Sclerostin

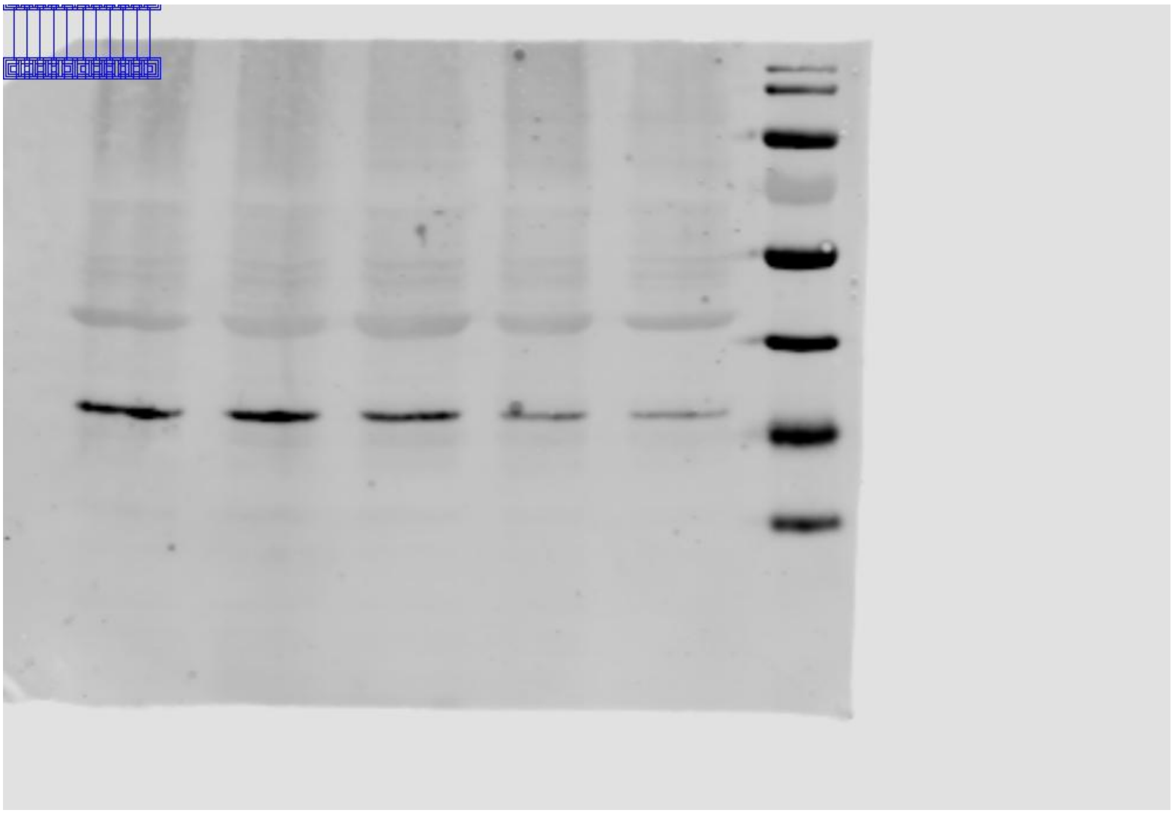

Figure 2 Expression of sclerostin in human breast cancer cell lines.
